# Novel Anti-Obesity Properties of *Palmaria mollis* in Zebrafish and Mouse Models

**DOI:** 10.3390/nu10101401

**Published:** 2018-10-02

**Authors:** Hiroko Nakayama, Yasuhito Shimada, Liqing Zang, Masahiro Terasawa, Kaoru Nishiura, Koichi Matsuda, Charles Toombs, Chris Langdon, Norihiro Nishimura

**Affiliations:** 1 Graduate School of Regional Innovation Studies, Mie University, Tsu, Mie 514-8507, Japan; 27293301@m.mie-u.ac.jp (H.N.); liqing@doc.medic.mie-u.ac.jp (L.Z.); nishimura.norihiro@mie-u.ac.jp (N.N.); 2 Mie University Zebrafish Drug Screening Center, Tsu, Mie 514-8507, Japan; 3 Department of Integrative Pharmacology, Mie University Graduate School of Medicine, Tsu, Mie 514-8507, Japan; 4 Department of Bioinformatics, Mie University Advanced Science Research Promotion Center, Tsu, Mie 514-8507, Japan; 5 Konan Chemical Manufacturing Co., Ltd., Yokkaichi, Mie 510-0103, Japan; terasawa@konanchemical.co.jp (M.T.); nisiura@konanchemical.co.jp (K.N.); matsuda@konanchemical.co.jp (K.M.); 6 College of Business, Oregon State University, Corvallis, OR 97331, USA; charles.toombs@oregonstate.edu; 7 Coastal Oregon Marine Experiment Station and Department of Fisheries and Wildlife, Hatfield Marine Science Center, College of Agricultural Sciences, Oregon State University, Corvallis, OR 97331, USA; chris.langdon@oregonstate.edu

**Keywords:** metabolic syndrome, animal model, comparative genomics, adipogenesis

## Abstract

(1) Background: The red seaweed *Palmaria mollis* (PM), which has a bacon-like taste, is increasingly being included in Western diets. In this study, we evaluate anti-obesity effects of PM using diet-induced obese (DIO) zebrafish and mice models. (2) Methods: We fed PM-containing feed to DIO-zebrafish and mice, and evaluated the anti-obesity effects We also analyzed gene expression changes in their liver and visceral adipose tissues (VAT). (3) Results: PM ameliorated several anti-obesity traits in both animals, including dyslipidaemia, hepatic steatosis, and visceral adiposity. In liver tissues of DIO-zebrafish and mice, PM upregulated gene expressions involved in peroxisome proliferator-activated receptor alpha (PPARA) pathways, and downregulated peroxisome proliferator-activated receptor gamma (PPARG) pathways, suggesting that the lipid-lowering effect of PM might be caused by activation of beta-oxidation and inhibition of lipogenesis. In VAT, PM downregulated genes involved in early and late adipocyte differentiation in zebrafish, but not in mice. (4) Conclusions: We have demonstrated that PM can prevent hepatic steatosis and visceral adiposity for the first time. Dietary supplementation of PM as a functional food may be suitable for obesity prevention and reduction in the prevalence of obesity-related diseases.

## 1. Introduction

Obesity is one of the most challenging public health problems in developed countries and it is of growing concern in developing countries. The prevalence of obesity has increased so rapidly that it has nearly doubled since 1980 and is considered to be a global epidemic [1]. Obesity increases the likelihood of various adverse health effects, particularly dyslipidemia, nonalcoholic hepatic disease, type 2 diabetes mellitus, cardiovascular diseases, and certain types of cancer. Dieting and physical exercise are the mainstays of treatment for obesity. If dieting and exercise are not effective, anti-obesity drugs may be taken to reduce appetite or inhibit fat absorption. However, most of these drugs are associated with side effects such as high blood pressure, restlessness, insomnia, and drug addiction [2]. For this reason, a variety of natural products have been studied for their potential to treat obesity with minimal side effects.

Seaweed, or marine macroalgae, is known for its high content of minerals and specific vitamins, but it also contains bioactive molecules such as polysaccharides, proteins, peptides, lipids, and polyphenols, making it a novel source of potential compounds for human health applications, including prevention of obesity [3]. For example, fucoxanthin, a marine carotenoid from brown seaweed suppressed weight gain and modulation of blood glucose and insulin levels in mice fed on a high-fat diet [4]. Fucoxanthinol, a metabolite of fucoxanthin, downregulated *peroxisome proliferator-activated receptor gamma* (*Pparg*) and exhibited a strong suppressive effect on adipocyte differentiation [5]. Alginates, one of the major dietary fibers in algae, are also well-known for their anti-obesity effects by increasing satiety, reducing energy intake and supporting weight reduction [6].

While consumption of whole seaweed is widely popular in Asia, limited numbers of consumers in several Western countries (e.g., Ireland, Scotland, Spain, France, Iceland, and Canada) use the red seaweed Atlantic dulse, *Palmaria palmata*. More recently, the closely related Pacific dulse, *Palmaria mollis* (PM), has been made popular on the US West Coast [7]. Dulse that tastes like bacon is a good source of minerals and vitamins compared with other vegetables, with a high protein content reaching around 20% of dry weight [8,9]. Recent studies have revealed that *P. palmata* possesses bioactive compounds that act as antioxidants [10], anti-inflammatory agents [11], blood pressure reducers [12], and inhibitors of cancer cell proliferation [13]. In addition, *P. palmata* extracts provided hydroxyl radical scavengers as well as stable free radical quenchers and inhibitors of lipid peroxidation [14]. Since obesity is associated with increases in endogenous lipid peroxidase [15,16], we hypothesized that consumption of the closely related Pacific dulse, PM would have therapeutic effects against obesity.

Over the last decade, the zebrafish (*Danio rerio*) has been increasingly employed in investigating human obesity and obesity-related diseases, including visceral adiposity [17,18], hepatic steatosis [19], atherosclerosis [20,21], and type 2 diabetes [22]. Overfed diet-induced obesity (DIO) zebrafish exhibit increased body weight gain, hypertriglyceridemia, hepatic steatosis [23,24], and visceral adiposity [25,26]. The organs that show signs of adiposity, such as fat accumulation in the liver and visceral adipose tissues, are similar to those found in humans. In addition, the pathophysiological pathways of visceral adiposity and hepatic steatosis are common to those found in obese humans [17,27]. Using DIO-zebrafish, we discovered that rhamnan sulphate from *Monostroma nitidum*, a green alga, attenuated body weight gain, dyslipidemia and hepatic steatosis through inhibition of lipogenesis [28].

In the present study, we investigated the anti-obesity effects of PM on body weight gain, hyperlipidemia, hepatic steatosis and visceral adiposity using DIO-zebrafish and mice. We further investigated the mechanisms by carrying out gene expression analysis in liver and visceral adipose tissues of both animal models.

## 2. Materials and Methods

### 2.1. Animals and Maintenance

All animal procedures were approved by the Ethics Committee of Mie University and were performed according to the Japanese animal welfare regulation ‘Act on Welfare and Management of Animals’ (Ministry of Environment of Japan) and complied with international guidelines. Zebrafish AB strain (The Zebrafish International Research Centre, Eugene, OR, USA) were maintained in our facility according to standard operational guidelines. NSY/HOS mice, a type 2 diabetes mellitus strain [29], were purchased from Hoshino Laboratory Animals (Saitama, Japan), and housed on a 12-h light/dark cycle at the Institute of Laboratory Animals at Mie University.

### 2.2. Zebrafish Experiments

PM powder was prepared by Konan Chemical Manufacturing Co. Ltd. (Yokkaichi, Mie, Japan). To prepare a zebrafish food containing 2.5% PM, we used gluten as a carrier material according to our previous study [30]. We decided on a PM dose of 2.5% (*w*/*w*) in zebrafish and mice experiments according to the dose used in other studies on algae-fed mice [31,32]. In addition, 1% (*w*/*w*) PM did not show any effect on body weight and blood chemistry in the zebrafish feeding experiment [33]. During feeding, the water flow to the experimental tanks was stopped for 2 h. Leftover food was removed once daily by vacuuming to avoid water pollution.

Experiment 1: this experiment was conducted according to protocols developed in our previous study with some modifications [34]. In brief, three-month-old female zebrafish were randomly divided, with 15 fish per 2 L tank, and fed during the first three weeks on a restricted ration of approximately 4 mg/fish/day using a Hikari Tropical Fancy Guppy diet (Kyorin, Hyogo, Japan). After this period of dietary restriction, zebrafish were randomly assigned into three treatment groups with five fish per 2 L tank (*n* = 10 per dietary group), and were overfed on a diet of newly hatched *Artemia* nauplii at a ration of approximately 150 cal/day [17]. As a control, we fed *Artemia* at a lower ration of 30 cal/day. After two weeks of overfeeding, fish were fed on a diet of *Artemia* supplemented with either 2 mg/fish PM-containing gluten granules (equal to 50 µg PM/fish) or only gluten granules every morning over a period of four weeks.

Experiment 2: three-month-old female zebrafish were randomly divided among treatments, as in Experiment 1. PM administration was started 1-week before DIO induction with a high calorific ration of *Artemia* nauplii supplemented with either PM-containing gluten granules or gluten granules alone (control) that was fed over an additional period of two weeks.

The body weights of zebrafish and feeding volumes of *Artemia* were measured weekly as previously reported [27].

### 2.3. Measurements of Plasma TG, LDL-C, T-CHO, and Fasting Blood Glucose in Zebrafish

At the end of the feeding experiment, zebrafish were deprived of food overnight to provide fasting levels of blood glucose, and blood was withdrawn from the dorsal artery by a heparinized glass capillary needle (GD-1, Narishige, Tokyo, Japan) as previously reported [35,36]. Blood glucose was measured using a hand-held glucometer (Glutest Neo Super, Sanwa Kagaku, Nagoya, Japan). The plasma levels of triacylglycerol (TG), low-density lipoprotein cholesterol (LDL-C) and total cholesterol (TCHO) were measured using Wako L-type TG, Wako L-type LDL-C, and Wako L-type TCHO (Wako Pure Chemicals, Osaka, Japan) assay kits according to the manufacturer’s protocol.

### 2.4. Oil Red O Staining of Zebrafish Liver

Liver tissues were collected from zebrafish by surgical manipulation under a SMZ745T stereoscopic microscope (Nikon, Tokyo, Japan). The preparation of liver sections and Oil Red O staining were performed as described previously [27]. Sections were also counterstained with Mayer’s hematoxylin (Wako Pure Chemicals, Osaka, Japan) to visualize the nuclei according to the manufacturer’s protocol. The Oil Red O-positive area was quantified using ImageJ software (Version 1.48d, National Institutes of Health, Bethesda, MD, USA) as previously reported [28].

### 2.5. Insulin Resistance in INS-EGFP Zebrafish

Tg (−1.0ins:EGFP) zebrafish were overfed for 10 days with or without PM feeding, as described previously [22]. The fish were then fasted overnight and anesthetized by placing them in a tank containing 500 ppm of 2-phenoxyethanol (Wako Pure Chemicals, Osaka, Japan). Enhanced green fluorescent protein (EGFP) signaling was captured using a BZ-X710 fluorescence microscope (Keyence, Tokyo, Japan). The EGFP intensity was quantified using ImageJ software (National Institutes of Health, Bethesda, MD, USA), as described in our previous study [22].

### 2.6. Mice Experiment

Six-month-old male NSY/HOS mice were assigned to three groups of six, housed individually, and fed on either the CE-7 normal diet (ND, CLEA Japan, Tokyo, Japan), high fat diet (Test Diet 58Y1; TestDiet, Richmond, IN, USA) or high fat diet (HFD) supplemented with PM (2.5% *w*/*w*) for four weeks to induce obesity. The compositions of ND and HFD are described in Table S1. During the feeding experiment, body weight and food intake were measured once per week. Mice were fasted for 14 h before blood sampling to provide fasting levels of blood glucose. The mice were euthanized with CO_2_ gas, then organ samples were taken and subsequently dissected for analysis.

### 2.7. Measurement of Liver Lipid in Mice

Liver tissues were collected by surgical manipulation, and fixed using 10% buffered formalin solution (Histo-Fresh, Pharma, Tokyo, Japan). Total lipids were extracted using a mixture of methyl *tert*-butyl ether (Wako Pure Chemical, Osaka, Japan) and methanol (Wako Pure Chemical, Osaka, Japan), as previously reported [37]. The dried lipid residues were dissolved in 20 µL of isopropanol containing 10% Triton X-100 (Nakarai Tesque, Kyoto, Japan). TG and TCHO were measured using Wako L-type TG and Wako L-type TCHO (Wako Pure Chemicals, Tokyo, Japan) assay kits according to the manufacturer’s protocol.

### 2.8. Computed Tomography

Zebrafish and mice were euthanized by immersion in an ice–water bath (5 parts ice/1 part water at ≤4 °C) for ≥20 min [38], and by over-anesthesia with isoflurane (Pfizer, Pearl River, NY, USA), respectively. Then 3D micro-CT scans were performed using an in vivo System R_mCT 3D micro-CT scanner (Rigaku, Tokyo, Japan) according to manufacturer’s instructions. The 3D images were reconstructed and viewed using i-View type R software (Version 1.50, J. Morita Mfg, Kyoto, Japan), and visualized and analyzed using CTAtlas Metabolic Analysis Ver. 2.03 software (Rigaku, Tokyo, Japan). In zebrafish, measurement of visceral adipose tissue volume was limited to the abdominal cavity, as previously reported [39].

### 2.9. RNA Extraction, cDNA Synthesis, and Quantitative Real-Time PCR

For total RNA extraction, liver and mesenteric adipose tissues of zebrafish and mice were immersed in RNAlater (Qiagen, Hilden, Germany), and store at 4 °C for several days. After bead-homogenization, total RNAs were isolated using a PureLink RNA mini kit (Life Technologies, Carlsbad, CA, USA). cDNA synthesis from 500 ng total RNA was performed using a ReverTra Ace qPCR RT Kit (Toyobo, Osaka, Japan). Quantitative real-time PCR (qPCR) was performed in cDNA samples using a Power SYBR Green Master Mix (Applied Biosystems, Foster City, CA, USA) and the ABI Stepone Plus Real-Time PCR System (Applied Biosystems, Foster City, CA, USA) in accordance with the manufacturer’s instructions. The sequences of the sense and antisense primers used for amplification are shown in Table S2. The relative mRNA expression levels were determined using endogenous standards of both glyceraldehyde-3-phosphate dehydrogenase (gapdh) and actin beta 1 (actb) for zebrafish as well as 18S ribosomal RNA (18S) for mice.

### 2.10. Statistical Analysis

All results were represented as means with their standard deviations (SD). Data were analyzed using Student’s *t*-test or one-way analysis of variance (ANOVA) with the Bonferroni–Dunn multiple comparison procedure, depending on the number of comparisons, using GraphPad Prism version 7 (GraphPad Software, San Diego, CA, USA). A *p*-value of less than 0.05 was considered statistically significant.

## 3. Results

### 3.1. PM Ameliorates Obese Phenotypes in Zebrafish (Experiment 1)

**Figure 1:**
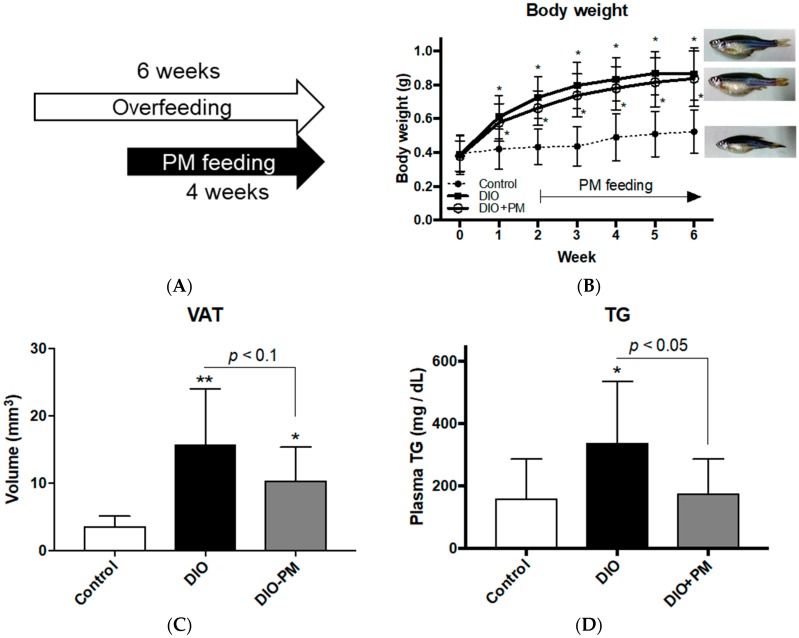

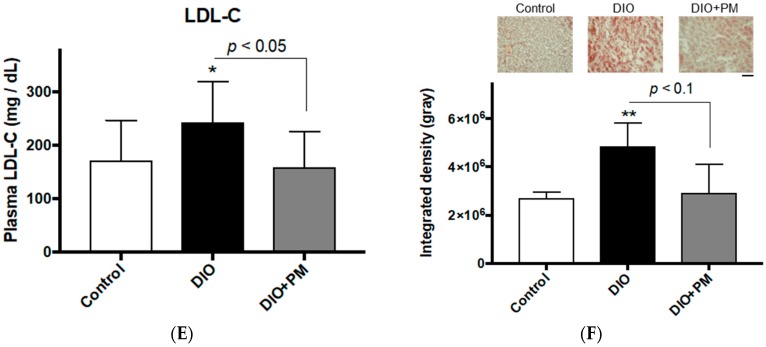
Post-administration of *Palmaria mollis* (PM) in overfed diet-induced obese (DIO)-zebrafish. (**A**) Schedule of PM supplementation in zebrafish Experiment 1. Overfeeding and PM supplementation started on week 0 and 2, respectively. (**B**) Body weight change during Experiment 1. (**C**) Volume of visceral adipose tissues (VAT) in week 6. (**D**–**E**) PM suppressed increases in plasma triacylglycerol (TG; **D**) and low-density lipoprotein cholesterol (LDL-C; **E**) in DIO-zebrafish. (**F**) Lipid accumulation in liver tissues. Lipid droplets (red spots) increased in DIO-zebrafish compared to control. Lower graph indicates the quantification of red signals. * *p* < 0.05, ** *p* < 0.01 vs. control. *n* = 7–10, error bars indicate standard deviations (SD).

After four weeks of PM administration in experiment 1 (Figure 1A), PM (DIO + PM) did not affect body weight compared to the overfed (DIO) group (0.86 ± 0.15 g in DIO vs. 0.84 ± 0.16 g in the DIO + PM group in week 6; Figure 1B). However, Computed tomography (CT) analyses showed a tendency (*p* < 0.1) for PM suppression of visceral adiposity in DIO-zebrafish in week 6 (15.8 ± 8.2 mm^3^ in DIO vs 10.4 ± 4.9 mm^3^ in the DIO + PM group; Figure 1C). Corresponding to the improvement of visceral adiposity, PM significantly (*p* < 0.05) suppressed plasma TG (337.3 ± 197.3 mg/dL in DIO vs. 176.2 ± 108.9 mg/dL in the DIO + PM group; Figure 1D) and LDL-C (243.1 ± 75.5 mg/dL in DIO vs. 158.4 ± 66.9 mg/dL in the DIO + PM group; Figure 1E) in week 6. Hyperlipidemia is typically accompanied by hepatic lipid deposition in obese [40], and we found that PM reduced lipid accumulation (Oil Red O staining) in the liver tissues more than in the DIO group (Figure 1F; *p* < 0.1). Food intake (Figure S1), T-CHO (Figure S2) and fasting blood glucose (FBG) levels (Figure S3) were not affected by PM administration.

### 3.2. PM Suppresses Early Stage of Obese Development in Zebrafish (Experiment 2)

**Figure 2:**
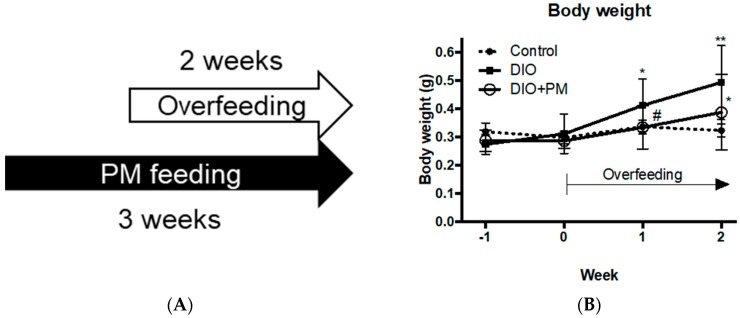

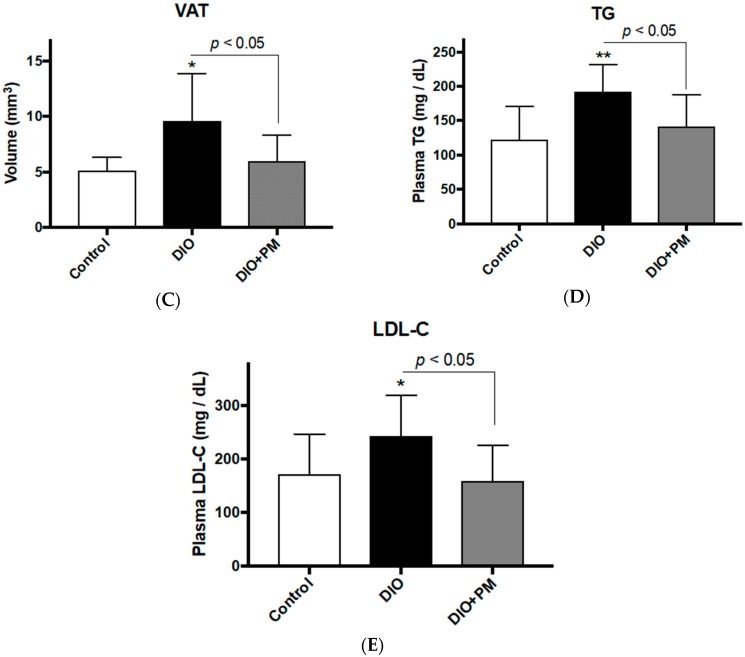
Pre-administration of PM in overfed DIO-zebrafish. (**A**) Schedule of PM supplementation in zebrafish Experiment 2. PM administration and overfeeding started on week –1 and week 0, respectively. (**B**) Body weight change during Experiment 2. (**C**) PM suppressed increases in visceral adipose tissues (VAT) in DIO-zebrafish in week 2. (**D**,**E**). PM suppressed increases in plasma triacylglycerol (TG; **D**) and low-density lipoprotein cholesterol (LDL-C; **E**) in DIO-zebrafish. * *p* < 0.05, ** *p* < 0.01 vs. control. # *p* < 0.05 between DIO and DIO + PM. *n* = 6–10, error bars indicate SD.

To evaluate the protective effect of PM against obese development, we conducted one-week PM administration before DIO induction (Figure 2A). For body weight, PM pre-administration significantly (*p* < 0.05) suppressed body weight increase compared to the DIO group in the first week overfeeding (0.41 ± 0.09 g in DIO vs. 0.33 ± 0.08 g in the DIO + PM group, Figure 2B). However, there was less significant difference after overfeeding for two weeks with three-weeks of PM administration (*p* < 0.1, 0.49 ± 0.13 g in DIO vs. 0.39 ± 0.13 g in the DIO + PM group). This result indicates that PM has a protective effect for body weight increase especially in the early phase of obese development. visceral adipose tissues (VAT) accumulation was strongly (*p* < 0.05) suppressed by PM (9.6 ± 4.3 mm^3^ in DIO vs 6.0 ± 2.3 mm^3^ in the DIO + PM group; Figure 2C), compared to the results of the “post-” PM administration (Experiment 1, Figure 1C, *p* < 0.1). In regards to blood chemistry, PM significantly (*p* < 0.05) suppressed levels of TG (192.9 ± 38.8 mg/dL in DIO vs. 141.8 ± 46.1 mg/dL in the DIO + PM group; Figure 2D) and LDL-C (182.5 ± 80.2 mg/dL in DIO vs. 103.8 ± 59.0 mg/dL in the DIO + PM group; Figure 2E), as was also observed in Experiment 1 (Figure 1D,E).

### 3.3. PM Ameliorates Obese Phenotypes in Mice

**Figure 3:**
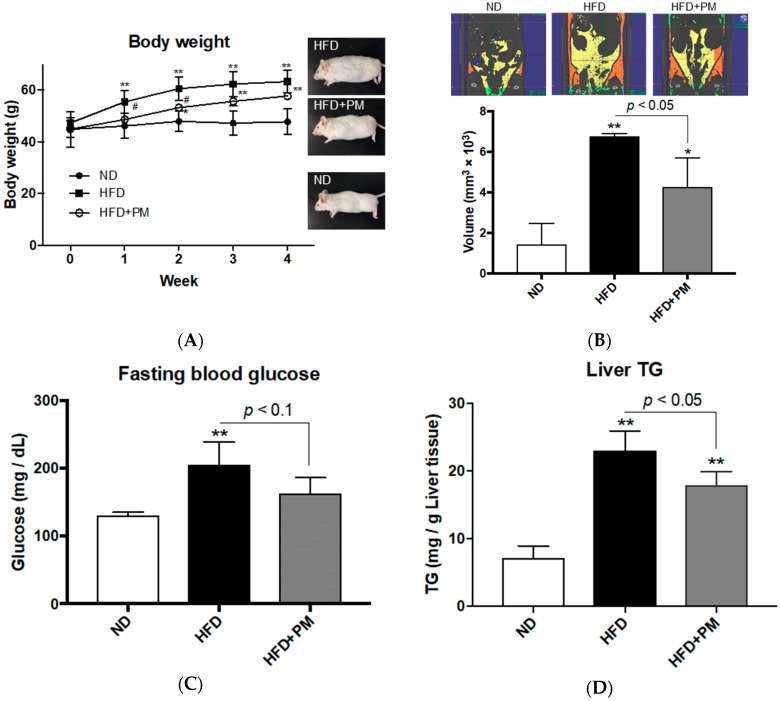
PM administration in NSY/HOS mice fed on a high fat diet. (**A**) NSY/HOS Mice fed on a high fat diet (HFD; closed squares) gained weight during the experiment. PM (open circles) suppressed body weight gain up to week 2. (**B**) Visceral adipose tissues (VAT) in week 6. HFD increased VAT compared to that of mice fed on a normal diet (ND), and PM significantly suppressed VAT increase. In the upper panel, yellow and orange colors indicate VAT and subcutaneous adipose tissues (SCAT), respectively. (**C**) Fasting blood glucose (FBG) levels in week 4. (**D**) PM suppressed liver TG increase in the HFD group. * *p* < 0.05, ** *p* < 0.01 vs. ND. # *p* < 0.05 between HFD and HFD + PM. *n* = 6, error bars indicate SD.

Whether the anti-obesity properties of PM are common among vertebrates, we next conducted a PM administration to mouse model for obesity. We chose NSY/HOS mice, a high fat diet (HFD) induced type 2 diabetic strain, to evaluate obese phenotypes that included body weight increase, visceral adiposity, hepatic steatosis and hyperglycemia. Four-weeks of a HFD feeding significantly (*p* < 0.01) increased body weight to about 1.3-times that of mice fed on the normal diet (ND, 63.4 ± 4.3 g in HFD vs. 47.8 ± 5.0 g in ND group), as previously reported [41]. PM administration (HFD + PM) significantly (*p* < 0.05) suppressed body weight increase in week 2 (*p* < 0.05, 60.6 ± 4.6 g in HFD vs. 53.3 ± 1.2 g in the HFD + PM group), while there was no significant difference between HFD and HFD + PM groups by week 4 (*p* = 0.17, 63.4 ± 4.3 g in HFD vs. 57.8 ± 1.3 g in the HFD + PM group; Figure 3A). This result seems to be the same as the results with zebrafish in Experiment 2 (Figure 2B) in that PM suppressed the early phase of body weight increase. There was no difference in food intake between HFD and HFD + PM groups (Figure S4). CT analysis showed that PM significantly suppressed (*p* < 0.05) the accumulation of VAT compared to that of the HFD group (6.8 ± 0.1 mm^3^ × 10^3^ in HFD vs. 4.3 ± 1.4 mm^3^ × 10^3^ in the HFD + PM group; Figure 3B), and these results were similar to those with zebrafish (Figure 1C and Figure 2C). For the volume of subcutaneous adipose tissues, there was no difference between HFD and HFD + PM groups (Figure S5). In regards to fasting blood glucose (FBG) levels, the HFD group showed significantly (*p* < 0.01) higher levels than those of the ND group as expected, because NSY/HOS mouse is a HFD-induced diabetic strain. HFD + PM group showed a tendency (*p* < 0.1) to reduce FBG levels compared to HFD group (205.2 ± 33.8 mg/dL in HFD vs. 163.0 ± 23.5 mg/dL in the HFD + PM group; Figure 3C). For hepatic lipid accumulation, PM significantly (*p* < 0.05) suppressed TG elevation (23.0 ± 2.9 mg/g tissue weight in HFD vs. 17.9 ± 2.0 mg/g in the HFD + PM group; Figure 3D), while TCHO levels were not significantly different between the experimental groups (Figure S6), similar to the results of DIO-zebrafish experiments (Figure 1D,F, Figure 2D and Figure S2). These results demonstrate that anti-obesity effects of PM occur in at least two different vertebrate taxa.

### 3.4. PM Effects on Gene Expressions Related to Lipid Metabolism in Liver Tissues

**Figure 4:**
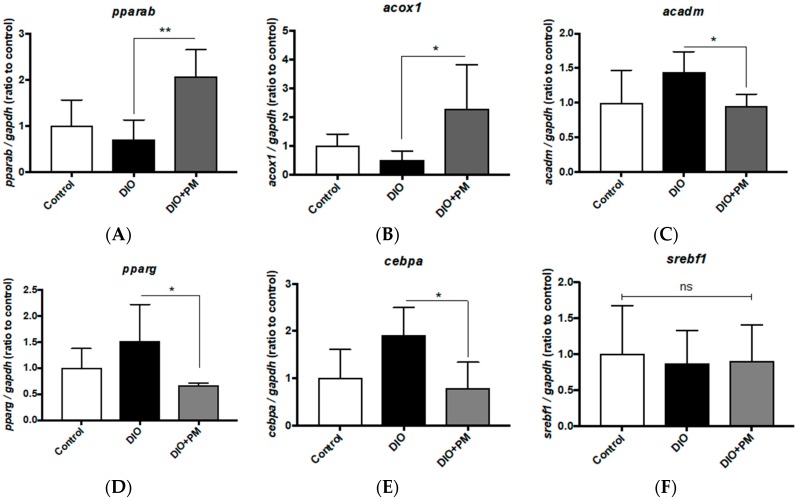
PM-induced gene expression changes in liver tissues of zebrafish. (**A**–**C**) PM effects on gene expression related to beta-oxidation in zebrafish. PM up-regulated *pparab* (**A**) and *acox1* (**B**) expressions, in contrast to the downregulation of *acadm* (**C**). (**D**–**F**) PM effects on gene expression related to lipogenesis in zebrafish. PM suppressed DIO-induced upregulation of *pparg* (**D**) and *cebpa* (**E**), not of *srebf1*. * *p* < 0.05, ** *p* < 0.01. *n* = 5, error bars indicate SD. *pparab*: *peroxisome proliferator-activated receptor alpha b*; gapdh: glyceraldehyde-3-phosphate dehydrogenase; *acox1*: *acyl-CoA oxidase 1, palmitoyl*; cebpa: *CCAAT/enhancer-binding protein alpha*; *acadm*: *acyl-CoA dehydrogenase*, *C-4 to C-12 straight chain*; *srebf1*: *sterol element binding transcription factor 1*.

In order to determine if the anti-obesity effects of PM depend on facilitation of lipolysis (beta-oxidation) or inhibition of lipogenesis, we analyzed gene expression profiles in the liver tissues of DIO-zebrafish and mice. In zebrafish liver, gene expressions related to beta-oxidation (which causes lipid clearance) such as *peroxisome proliferator-activated receptor alpha b* (*pparab*), and its target gene, *acyl-CoA oxidase 1, palmitoyl* (*acox1*), were significantly (*p* < 0.01 and *p* < 0.05 vs. DIO group) upregulated by PM administration (Figure 4A,B). In contrast to *acox1*, another pparab-target gene, *acyl-CoA dehydrogenase, C-4 to C-12 straight chain* (*acadm*), was suppressed (*p* < 0.05) by PM (Figure 4C). For lipogenic genes, expression levels of *peroxisome proliferator-activated receptor gamma* (*pparg*), and its downstream gene, *CCAAT/enhancer-binding protein alpha* (*cebpa*), were also downregulated by PM supplements (Figure 4D,E, *p* < 0.05 vs. DIO), while another pparg-downstream gene, *sterol element binding transcription factor 1* (*srebf1*), was not affected (Figure 4F). These results indicate that PM would promote beta-oxidation and suppress lipogenesis in the zebrafish livers.

**Figure 5:**
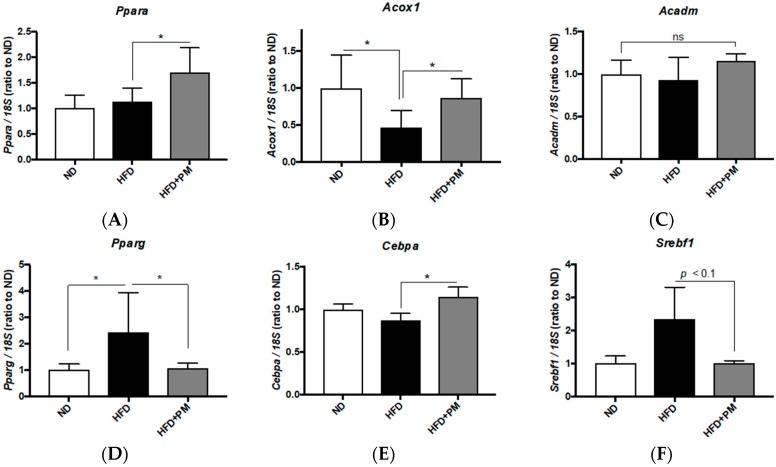
PM-induced gene expression changes in liver tissues of mice. (**A**–**C**) PM effects on gene expression related to beta-oxidation in mice. PM upregulated *Ppara* (**A**) and *Acox1* (**B**) expression, as seen with zebrafish (Figure 4A,B). *Acadm* (**C**) expression was not affected by PM. * *p* < 0.05. *n* = 6. (**D**–**F**) PM effects on gene expression related to lipogenesis in zebrafish. PM suppressed HFD-induced upregulation of *Pparg* (**D**), with increase of *Cebpa* expression (**E**). *Srebf1* was slightly (*p* < 0.1) downregulated by PM (**F**). * *p* < 0.05. *n* = 6, error bars indicate SD. ns indicates no significant difference.

In mice liver tissues, PM significantly (*p* < 0.05) upregulated *Ppara* (Figure 5A) and *Acox1* (Figure 5B) expression, while *Acadm* was not increased (Figure 5C). This is agreeing with the results of the zebrafish experiments (Figure 4A–C) and implies that PM would promote beta-oxidation also in mouse liver. For lipogenesis genes, *Pparg* expression was significantly (Figure 5D, *p* < 0.05) downregulated by PM, as seen in the zebrafish experiments; however, *Cebpa* expression was slightly (1.3-fold, *p* < 0.05) upregulated (Figure 5E), in contrast to *cebpa* downregulation observed with zebrafish (Figure 4E). On the other hand, PM showed a tendency (*p* < 0.1) to suppress *Srebf1* expression in mice liver (Figure 5F).

### 3.5. Different Transcriptional Responses in VAT between Zebrafish and Mice

**Figure 6:**
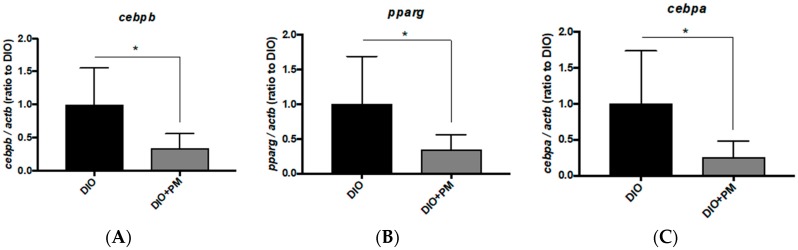

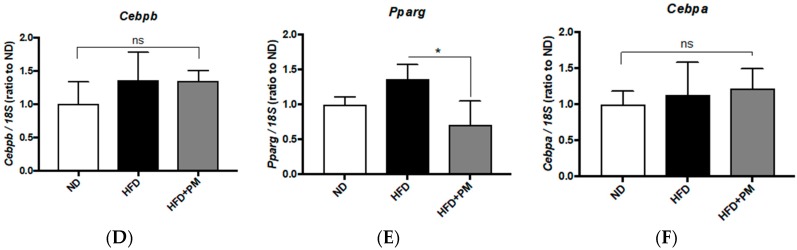
PM-induced gene expression changes in VAT of zebrafish and mice. (**A**–**C**) PM effects on gene expression related to adipocyte differentiation in zebrafish. PM downregulated *cebpb* (**A**), *pparg* (**B**) and *cebpa* (**C**) expression. (**D**–**F**) PM effects on gene expression related to adipocyte differentiation in mice. PM did not affect *Cebpb* (**D**) and *Cebpa* expression (**F**), while *Pparg* expression (**E**) was suppressed by PM compared to HF group. * *p* < 0.05. *n* = 6, error bars indicate SD.

PM suppressed VAT accumulation in zebrafish (Figure 1C) and mice (Figure 3B). To elucidate PM’s mechanism on VAT in zebrafish and mice, we conducted qPCR analysis of genes involved in adipogenesis and lipid metabolism. In zebrafish VAT, the early adipogenesis marker CCAAT/enhancer-binding protein beta (*cebpb*), was significantly (*p* < 0.05) suppressed by PM (Figure 6A) as well as downregulation of the late differentiation markers *pparg* (Figure 6B) and *cebpa* (Figure 6C). These results indicate that PM suppressed adipocyte differentiation during obesity development in zebrafish VAT.

In mouse VAT, expression of *Pparg* was significantly (*p* < 0.05) downregulated by PM administration (Figure 6E), similar to zebrafish results (Figure 6B); however, other marker genes, *Cebpb* (Figure 6D) and *Cebpa* (Figure 6F) were not changed. In addition, we found that expression of the *Pparg*-downstream *Srebf1* was slightly (*p* < 0.1) downregulated by PM in mouse VAT, which was not changed in zebrafish (Figure S7). These results indicate that anti-VAT mechanisms of PM in mouse were different from those in zebrafish, especially in respect to adipocyte differentiation.

## 4. Discussion

### 4.1. Anti-Obesity Mechanisms of PM in Zebrafish and Mice Models of Obesity

In this study, we found that Pacific dulse, *Palmaria mollis* (PM), showed anti-obesity effects in diet-induced obesity models. There were some differences between zebrafish and mice in how PM exerts anti-obesity mechanisms. In liver, lipid metabolism in zebrafish is very similar to that in humans in terms of lipid synthesis and fatty acid oxidation [42]. Recent studies have begun to explore zebrafish homologues of mammalian genes involved in lipid metabolism; for example, several transgenic and mutant zebrafish models with hepatic steatosis exhibit elevated expression of important lipogenic genes, such as *cebpa*, *pparg*, *srebp1*, and *acetyl-CoA carboxylase 1* (*acc1*), which show a similar mechanism to that observed in human hepatic steatosis [43]. In this study, transcriptional responses of liver tissues of animals fed on PM were quite similar between zebrafish and mice, in activation of beta-oxidation and suppression of lipogenesis (Figure 4 and Figure 5); however, there was little difference in peroxisome proliferator-activated receptor alpha (PPARA)- and peroxisome proliferator-activated receptor gamma (PPARG)-downstream pathways. In addition to the difference in ACADM response of PPARA-driven beta-oxidation pathway (Figure 4C in zebrafish and Figure 5C in mice), PPARA-driven lipogenic downstream CEBPA (Figure 4E and Figure 5E) and SREBF1 (Figure 4F and Figure 5F) showed different responses to PM between these species. Furthermore, PM-induced gene expression alternation in VAT were apparently different between zebrafish and mice. In zebrafish, PM suppressed adipocyte differentiation from pre-adipocytes, while did not in mouse VAT (Figure 6). Whereas, in vitro gene expression pathways of adipocyte differentiation and maturation of mouse 3T3-L1 preadipocytes are quite similar to those of zebrafish [26], PM administration would likely only suppress lipid synthesis in adult mice (*Srebf1* down-regulation in Figure S7). This consequently blocks hypertrophy of white adipocytes in mice, because they have already differentiated adipocytes, similar to those in humans. We assume that we can choose animal models for different purposes, for example, zebrafish as an in vivo model for identifying inhibitors of adipocyte differentiation.

In addition, PM showed a slight (*p* < 0.1) FBG-lowering effect in DIO-mice (Figure 3C), but not in zebrafish (Figure S3). We hypothesized that PM would also improve insulin resistance in zebrafish, although the efficacy of PM was not enough to reduce FBG. To examine this, we performed a PM-feeding experiment with overfed Tg (−1.0ins:EGFP) zebrafish, which visualized insulin production as an EGFP signal driven by the zebrafish preproinsulin promoter [44]. In accordance with our previous result [22], overfeeding significantly increased the insulin-EGFP signal compared to normal feeding (Figure S8). PM feeding in overfed zebrafish slightly (*p* < 0.1) suppressed the increase in EGFP signals, suggesting that PM administration at a higher dose (>2.5% *w*/*w*) could improve insulin resistance in obese animals.

### 4.2. Possible Anti-Obesity Constituents in PM

Some Asian red algae have already been reported to have anti-obesity effects; for example, the extracts of *Plocarmium telfaireaes* (PTE), a Korean red alga, inhibited adipogenesis of mouse pre-adipocyes with downregulation of *Pparg*, *Cebpa*, and *Srebf1* [45]. PTE also suppressed body weight increase and visceral adiposity in obese mice (male C57BL/6) fed a high-fat diet. The results with PTE seem quite similar to our results with PM; however, in addition to the effects against visceral adiposity, PM also ameliorated hepatic steatosis and hypertriglyceridaemia through different mechanisms. This implies that PM possesses multifunctional properties and bioactive molecules against obesity. In the previous studies, *Palmaria palmata*, a closely related species to PM, was shown to contain several bioactive molecules or peptides that promote human health; for example, dipeptidyl peptidase 4 (DPP4) inhibitory peptides [46] and angiotensin I converting enzyme (ACE) inhibiting peptides [47] were discovered recently. DPP4 inhibition is an established therapeutic approach for diabetes, and PM slightly (*p* < 0.1) suppressed FBG elevation in our mice study (Figure 3C). In addition to improvement of insulin resistance, DPP4 inhibitor ameliorated visceral adiposity via inhibition of adipogenesis by partial enhancement of energy expenditure along with metabolic changes in DIO-mice [48]. ACE inhibition is a standard approach against hypertension. A recent study showed that co-administration of ACE inhibitor enalapril and resveratrol improved glucose and lipid mice profiles by modulating expression of some lipogenesis genes by decreasing mRNA expression of fatty acid synthase (*Fasn*), acetyl-CoA carboxylase alpha (*Acaca*), and *Pparg* [49] in a way that is similar to the results of our study (Figure 4 and Figure 5). Their results indicate that these inhibitory peptides are ones of the strong candidates for anti-obese constituents also in PM. Of course, there is a possibility that PM contains other anti-obesity compounds, such as carotenoid fucoxantin and fucoxanthinol, that are similar to those found in other seaweeds. These carotenoids and their metabolites downregulate *Pparg* and exhibit strong suppressive effects on adipocyte differentiation in obese mice [5]. Further studies are needed to elucidate anti-obesity constituents in PM.

### 4.3. A Perspective for Clinical Use of PM against Human Obesity

Contrary to our promising results in DIO-animals, Allsopp et al. recently reported that consuming *P. palmata*-enriched bread stimulated inflammation, and increased serum TG and thyroid stimulating hormone (TSH) in a healthy (non-obese) people [50]. High level of iodides in *P. palmata* would stimulate TSH synthesis and secretion from thyroids, leading to enhanced inflammation. The authors stated that these effects were not likely to impact health as levels remained within the normal clinical range, however, these phenomena can happen also in PM administration to obese people. As chronic inflammation in obese subjects is highly associated with metabolic syndrome and other obesity-related diseases, including cancer and circadian rhythm disturbance [51], it is necessary to observe the long-term effects of PM feeding. Interestingly, Allsop et al. also described that the hot water extract of *P. palmata* promoted free glycerol release from mouse 3T3-L1 adipocytes (Figure 4 in their paper), suggesting the induction of adipolysis by *P. palmata* uptake. This result was similar to our in vivo results of PM administration (Figure 1, Figure 2 and Figure 3). These results indicate that PM can be used as anti-obese natural product to human with acceptable side effects.

## 5. Conclusions

Pacific dulse, *Palmaria mollis* (PM), is a seaweed species consumed in the Western world. We demonstrated that oral administration of PM ameliorated hepatic steatosis and visceral adiposity in zebrafish and mice models of obesity. The therapeutic mechanisms of PM were dependent on the model species; however, gene expression analysis revealed that PM suppressed beta-oxidation and lipid synthesis in the livers of both species. This is the first study to demonstrate PM’s anti-obesity properties in vivo. Our findings support that dietary supplementation of PM as a functional food may have important implications for the prevention of obesity and its-related diseases.

## Supplementary Materials

The following are available online at http://www.mdpi.com/2072-6643/10/10/1401/s1, Figure S1. Food intake in zebrafish Experiment 1; Figure S2. Plasma total cholesterol (THO) levels in zebrafish Experiment 1; Figure S3. Fasting blood glucose (FBG) levels in zebrafish Experiment 1; Figure S4. Food intake during the mouse experiment; Figure S5. Subcutaneous adipose tissues (SCAT) in week 4 in the mouse Experiment; Figure S6. Liver total cholesterol (TCHO) levels in the mouse experiment, Figure S7. PM effects on Srebf1 expression in zebrafish and mouse VAT, Figure S8. PM effects on insulin-EGFP expression in zebrafish; Table S1. Food compositions of control diet (ND) and high fat diet (HFD) in mice; Table S2. Primer sequences for qPCR.
