# Supplementary material for: Novel Anti-Obesity Properties of Palmaria mollis in Zebrafish and Mouse Models
Source: Nutrients. 2018 Oct 2;10(10):1401. doi: 10.3390/nu10101401 (PMC6213011; doi:10.3390/nu10101401)
Supplement: Supplementary file 1 [file nutrients-10-01401-s001.docx]

Article

A novel anti-obesity properties of *Palmaria mollis* in zebrafish and mouse models.

Hiroko Nakayama ^1,†^, Yasuhito Shimada ^2-4,^*^,†^, Liqing Zang ^1^, Aya Kuwayama ^1^, Masahiro Terasawa ^5^ , Kaoru Nishiura ^5^, Koichi Matsuda ^5^, Charles Toombs ^6^, Chris Langdon ^7^ and Norihiro Nishimura ^1,2^

Supplemental materials

Supplemental Figures and Tables

**Figure S1.** Food intake in zebrafish Experiment 1. ***p* < 0.01 vs. control. *n* = 6, error bars indicate SD.

**Figure S2.** Plasma total cholesterol (THO) levels in zebrafish Experiment 1. *n* = 10, error bars indicate SD.

**Figure S3.** Fasting blood glucose (FBG) levels in zebrafish Experiment 1. *n* = 10, error bars indicate SD.

**Figure S4.** Food intake during the mouse Experiment.

**Figure S5.** Subcutaneous adipose tissues (SCAT) in week 4 in mouse Experiment. ***p* < 0.01 vs. ND group. *n* = 6, error bars indicate SD.

**Figure S6.** Liver total cholesterol (TCHO) levels in mouse Experiment. *n* = 6, error bars indicate SD.

|  |  |
| --- | --- |

**Figure S7.** PM effects on Srebf1 expression in zebrafish and mouse VAT. *n* = 6, error bars indicate SD.


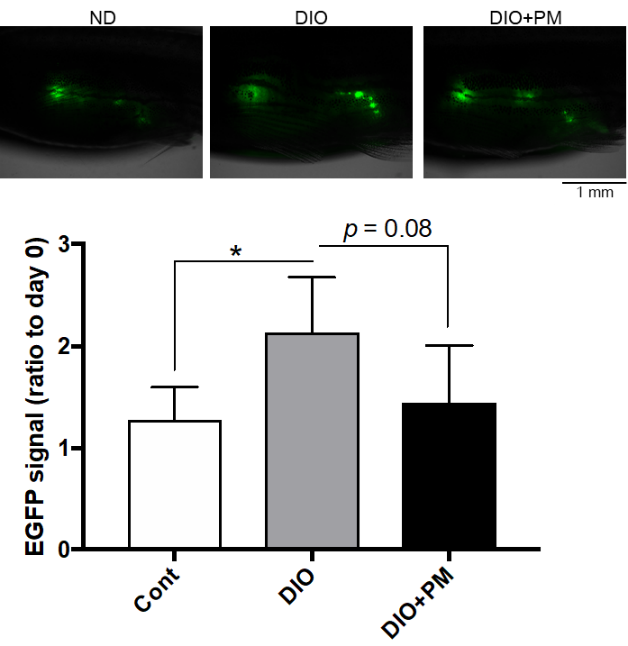


**Figure S8.** PM effects on insulin-EGFP expression in zebrafish. **p* < 0.05, *n* = 5, error bars indicate SD.

**Table S1.** Food compositions of control diet (ND) and high fat diet (HFD) in mouse Experiment.

|  | **ND**  (CE-7) | **HFD**  (58Y1) |
| --- | --- | --- |
| Protein | 17.9% | 23.1% |
| Fat | 3.7% | 34.9% |
| Fiber | 5.1% | 6.5% |
| Carbohydrate | 58.9%^1^ | 25.9% |
| Ash | 6.4% | 3.8% |
| Water | 8.1% | 5.7%^1^ |
|  |  |  |
| Energy (kcal/g) | 3.4 | 5.1 |

^1^ Calculated from other components.

**Table S2.** Primer sequences for qPCR.

| **Zebrafish** | |  |  |  |  |  |
| --- | --- | --- | --- | --- | --- | --- |
|  | Symbol | Gene name | Forward primer sequence (5' - 3') | Reverse primer sequence (5' - 3') | Product size (bp) | Locus ID |
|  | acadm | acyl-Coenzyme A dehydrogenase, medium chain | TGGAGAAGGAGCTGGCTTTA | AAGACACTGCCTGGTGCTCT | 170 | NM_213010 |
|  | acox1 | acyl-Coenzyme A oxidase 1, palmitoyl | ACAGCACAGCAAGAGTAACG | TGAAGGGCATAAAGCAGAGC | 177 | NM_001005933 |
|  | srebf1 | sterol regulatory element binding transcription factor 1 | CAGAGGGTGGGCATGCTGGC | CAGAGGGTGGGCATGCTGGC | 118 | NM_001105129 |
|  | cebpa | CCAAT/enhancer binding protein (C/EBP), alpha | AACGGAGCGAGCTTGACTT | AAATCATGCCCATTAGCTGC | 250 | NM_131885 |
|  | cebpb | CCAAT/enhancer binding protein (C/EBP), beta | CGACTTTCTCTCTGAGGGGAACA | CAGCGAGATGTAGTTCTTGTAGTTCT | 44 | NM_131884.2 |
|  | gapdh | glyceraldehyde-3-phosphate dehydrogenase | GTGGAGTCTACTGGTGTCTTC | GTGCAGGAGGCATTGCTTACA | 152 | NM_001115114 |
|  | pparab | peroxisome proliferator-activated receptor alpha b | CGTCGTCAGGTGTTTACGGT | AGGCACTTCTGGAATCGACA | 250 | NM_001102567 |
|  | pparg | peroxisome proliferator activated receptor gamma | CTGCCGCATACACAAGAAGA | TCACGTCACTGGAGAACTCG | 152 | NM_131467 |
|  |  |  |  |  |  |  |
|  |  |  |  |  |  |  |
| **Mouse** | |  |  |  |  |  |
|  | Symbol | Gene name | Forward primer sequence (5' - 3') | Reverse primer sequence (5' - 3') | Product size (bp) | Locus ID |
|  | 18S | 18S ribosomal RNA | GGCCGTTCTTAGTTGGTGGAGCG | CTGAACGCCACTTGTCCCTC | 133 | NR_003278 |
|  | Acadm | acyl-Coenzyme A dehydrogenase, medium chain | AGTACCCGTTCCCTCTCATCA | CCATACGCCAACTCTTCGGTAA | 108 | NM_007382 |
|  | Acox1 | acyl-Coenzyme A oxidase 1, palmitoyl | CAGGAAGAGCAAGGAAGTGG | CCTTTCTGGCTGATCCCATA | 169 | NM_015729 |
|  | Cebpa | CCAAT/enhancer binding protein (C/EBP), alpha | GGACAAGAACAGCAACGAGTACC | CGGTCATTGTCACTGGTCAACT | 113 | NM_007678 |
|  | Cebpb | CCAAT/enhancer binding protein (C/EBP), beta | CGCAACCTGGAGACGCAGCA | GGCTCGGGCAGCTGCTTGAA | 108 | NM_001287739 |
|  | Hprt | hypoxanthine guanine phosphoribosyl transferase | ATACAGGCCAGACTTTGTTGG | CAACTTGCGCTCATCTTAGG | 134 | NM_013556 |
|  | Ppara | peroxisome proliferator-activated receptor alpha | TGCAAACTTGGACTTGAACG | AGGAGGACAGCATCGTGAAG | 60 | NM_011144 |
|  | Pparg | peroxisome proliferator activated receptor gamma | TGTGGGGATAAAGCATCAGGC | CCGGCAGTTAAGATCACACCTAT | 97 | NM_001127330 |
|  | Srebf1 | sterol regulatory element binding transcription factor 1 | TCCAGTGGCAAAGGAGGCAC | CAGCATGCTCATTCGCTGCC | 118 | NM_011480 |
